# On the Treatment of Croup by Calomel and Alum

**Published:** 1850-12

**Authors:** 


### RECORD OF MEDICAL SCIENCE.

### PATHOLOGY AND PRACTICE OF MEDICINE.

*On the Treatment of Croup by Calomel and Alum.* By M. Miguel.—In a letter addressed to the Medical Society of Inde-et-Loire, M. Miguel of Torres, has given some important details of the result of the method of treatment which he employs in diptheritis.

He relates that, about twelve years ago, a little girl, seven years old, having been simultaneously seized with angina and croup, he proposed to perform the operation of tracheotomy, which was objected to by the parents. Being thus deprived of the last resource of art, he alternately administered to the child, every hour, two grains of calomel and three grains of alum. This treatment was continued a week, and produced no purgation nor salivation. Since this case, M. Miguel has treated twenty-six cases of croup, only three or four of which were doubtful; and only five cases have been fatal. He attributes the efficacy of his method to the mercury; but as it is liable to produce salivation and other disastrous consequences, these must be prevented; and M. Miguel thinks he has attained this object by combining alum with calomel. He thinks that when the calomel and alum are alternated, the latter serves to circumscribe the mercurial action, which should also be well watched, so that the administration of calomel may be suspended on the least appearance of mercurial toxication.

*Remarks.*—The treatment of M. Miguel is founded on the property which is attributed to mercury, of diminishing the plasticity of the blood, and opposing the formation of false membranes. The important point is, that this treatment has proved successful in a certain number of cases. It should be known also that such treatment will not supercede the necessity of emetics and of energetic cauterization, when the disease has commenced in the pharynx. In such cases mercury alone is useless; it may be prescribed, but the local treatment is that which must be chiefly depended on, to arrest the progress of the disease. Of this we saw a remarkable instance some time ago in the practice of M. Trousseau.

In this case, the diphtheritis had commenced on the tonsils, and had extended towards the larynx. On the first day of its appearance, an emetic of sulphate of copper was administered, and the back of the throat was well cauterized with fuming hydrochloric acid. This cauterization was repeated once on the next day, twice on the day following, and once on the subsequent day. At the same time the patient took, in small quantities every quarter or half hour, a mixture of ten grammes of alum with the same quantity of honey. This is preferable to alum in powder, because the medicine comes into permanent contact with the throat and arytænoepiglottic cartilages.

In prescribing alum, M. Trousseau used it as an auxilliary to cauterization, not as a corrective to mercury, which he did not give during the whole progress of the case. From the third day there was a steady improvement; and on the sixth, the cough had lost its croupy character. The voice continued rather weak, which showed the existence of false membranes on some points. Cauterization was continued once a day, for two days, together with the alum and honey: and the child recovered. This method of treatment is considered, by M. Trousseau, to be the most certain which can be employed in cases of croup.—*Dub. Med. Press., from Journal de Méd.*

